# Patent Ductus Arteriosus in Preterm Neonates: A 2025 Synthesis of Evidence‐Based Paradigms and Emerging Clinical Strategies

**DOI:** 10.1002/pdi3.70032

**Published:** 2025-12-24

**Authors:** Aditya Hemendra Bhatt, Somashekhar Marutirao Nimbalkar, Dipen Vasudev Patel, Reshma Kushal Pujara

**Affiliations:** ^1^ Department of Neonatology Pramukhswami Medical College, Bhaikaka University Karamsad Gujarat India

## 1 Introduction

The American Academy of Pediatrics (AAP) recently published an updated clinical report on the management of patent ductus arteriosus (PDA) in preterm infants authored by Ambalavanan, Aucott, Salavitabar, Levy, and the Committee on Fetus and Newborn and Section on Cardiology and Cardiac Surgery [1]. This comprehensive report addresses the ongoing controversies and uncertainties surrounding PDA management, providing evidence‐based guidance for clinicians caring for preterm infants (Table 1).

**TABLE 1:** Summary of key updates in 2025 American Academy of Pediatrics clinical consensus report.

Aspect	Previous approach (2016) [2]	2025 update [1]	Evidence basis
Prophylactic treatment	Considered in some centers	Strongly discouraged	138 RCTs showing no mortality/BPD benefits
Fluid restriction	Recommended for PDA prevention	Limited to the early period (no post‐Dx benefit)	Historical data with modern immaturity gaps
First‐line medical therapy	Indomethacin preferred	Ibuprofen is favored over indomethacin	Lower NEC risk (RR 0.68)
Transcatheter closure	Limited to > 6 kg infants	Approved for infants ≥ 700 g	Piccolo Occluder safety data
Surgical ligation rates	4.4% in 2014	0.84% in 2021	National database trends
Expectant management	Limited adoption	Recommended default approach	Baby‐OSCAR/BeNeDuctus trial results

Abbreviations: Baby‐OSCAR, Outcome after Selective early treatment for Closure of patent ductus ARteriosus in preterm infants; BPD, bronchopulmonary dysplasia; Dx, diagnosis; NEC, necrotizing enterocolitis; PDA, patent ductus arteriosus; RCT, randomized controlled trial; RR, relative risk.

## 2 Understanding PDA in Preterm Infants

The ductus arteriosus is a fetal blood vessel that typically closes shortly after birth in term infants. However, in preterm infants, especially those born at extreme prematurity, this closure is often delayed. The report highlights that spontaneous closure rates vary significantly by gestational age: whereas the ductus remains open at 4 days in only 10% of infants born at 30–37 weeks, it persists in more than 90% of those born at 24 weeks gestation [1]. This clinical challenge is particularly complex when a PDA becomes “hemodynamically significant” (hsPDA) and requires intervention. The persistence of a PDA can lead to increased pulmonary blood flow and potential “ductal stealing” from systemic circulation, which may contribute to complications, including bronchopulmonary dysplasia, necrotizing enterocolitis, and intraventricular hemorrhage [1]. However, the report emphasized that these associations do not necessarily prove causation, which partly explains the ongoing debate about optimal management strategies.

## 3 Assessment of Hemodynamic Significance

One of the most valuable aspects of the report by Ambalavanan and colleagues are the authors of 2025 clinical report mentioned in [1] is its detailed guidance for assessing the hemodynamic significance of a PDA. The authors acknowledge that there exists no single definition of hsPDA, but they provide a practical framework combining clinical and echocardiographic criteria. Echocardiography remains the cornerstone of PDA assessment, evaluating not only the presence of a PDA but also its hemodynamic impact on pulmonary and systemic circulation. The report details specific measurements, including the left atrial‐to‐aortic root ratio, PDA diameter, flow patterns, and effects on end‐organ perfusion. The authors also discuss the potential role of biomarkers, such as B‐type natriuretic peptide (BNP), while noting their limitations.

## 4 Evidence‐Based Management Approaches

Groundbreaking studies published prior to the 2025 AAP report include the PDA‐TOLERATE [3], BeNeDuctus [4] and Baby‐OSCAR [5] trials (Table 2). Perhaps the most significant contribution of this report is its thorough review of management strategies on the basis of current evidence. Several key recommendations emerge as follows:

1. Prophylactic interventions are not recommended. The report clearly states that prophylactic medical interventions (those not guided by PDA status) are not recommended at any age or birth weight as they have no effect on mortality, bronchopulmonary dysplasia (BPD), or neurodevelopmental impairment [1].
2. Early routine treatment is not beneficial. Very early (< 72 h) or early (< 7–14 days) routine treatment to induce PDA closure, regardless of hemodynamic significance, does not improve outcomes and is not recommended.
3. Conservative management is often appropriate. The report acknowledges the growing trend toward conservative (expectant) management, allowing for spontaneous closure without intervention in many cases. This approach appears safe on the basis of retrospective data and recent randomized trials.
4. Selective treatment may be considered after 2 weeks. For infants with a hemodynamically significant PDA beyond 2 weeks of age, pharmacologic closure may be reasonable, with ibuprofen generally preferred over indomethacin or acetaminophen owing to its safety profile (Table 3).
5. Procedural closure for persistent hsPDA. If the hsPDA persists despite medical therapy (or if medical therapy is contraindicated), transcatheter closure or surgical ligation may be considered. Table 4 provides the comparative assessments of various treatment modalities.

**TABLE 2:** Comparison of major PDA clinical trials.

Parameter	Baby‐OSCAR (2024)	PDA‐TOLERATE (2019)	BeNeDuctus (2023)
Design	Multicenter RCT (UK)	International RCT (17 sites)	European RCT (17 NICUs)
Population	23–28^+^^6^ weeks GA	< 28 weeks GA	< 28 weeks GA
Sample size	653 infants	202 infants	273 infants
Intervention timing	≤ 72 h postnatal age	6–14 days postnatal age	24–72 h postnatal age
Treatment arms	Ibuprofen versus placebo	Early routine versus conservative	Expectant versus early ibuprofen
Primary outcome	Death/BPD at 36 w PMA	Ligation/PDA at discharge	NEC/BPD/death at 36 w PMA
Key findings	69.2% versus 63.5% (NS, *p* = 0.1)	No difference in composite outcomes	46.3% versus 63.5% (non‐inferiority met)
Notable risks	↑ Trend in mortality (13.6% vs. 10.3%)	↑ Late‐onset sepsis in early group	↑ BPD in ibuprofen group (50.9%)
Practice impact	Supported conservative management	Validated delayed treatment approach	Established expectant management

Abbreviations: AAP, American Academy of Pediatrics; Baby‐OSCAR, Outcome after Selective early treatment for Closure of patent ductus ARteriosus in preterm infants; BeNeDuctus, Best treatment of patent ductus arteriosus in preterm infants; BPD, bronchopulmonary dysplasia; Dx, diagnosis; GA, gestational age; NEC, necrotizing enterocolitis; NICU, neonatal intensive care unit; NS, not significant; PDA, patent ductus arteriosus; PDA‐TOLERATE, Treatment of patent ductus arteriosus at one week of age; PMA, post‐menstrual age; RCT, randomized controlled trial; UK, United Kingdom.

**TABLE 3:** Pharmacologic treatment recommendation comparison.

Agent	2016 American Academy of Pediatrics clinical consensus report	2025 American Academy of Pediatrics clinical consensus report	Closure efficacy
Ibuprofen	Alternative to indomethacin	First‐line for ≥ 26 weeks GA	70.3% (standard dose)
Acetaminophen	Not addressed	Alternative with hepatic monitoring	83.6% efficacy in recent studies
Indomethacin	Preferred agent	Restricted due to NEC risk	Similar closure but complications ↑
Repeat courses	Limited data	Max 2 courses recommended	↓ Efficacy after the 2nd course

Abbreviations: GA, gestational age; NEC, necrotizing enterocolitis.

**TABLE 4:** Long‐term outcome comparison.

Outcome	Medical closure	Transcatheter	Surgical
BPD	No difference versus placebo	↓ Ventilation duration	↑ Risk (OR 1.32)
Neurodevelopment	No measurable benefit	Insufficient data	↑ Cerebral palsy risk
Mortality	Neutral	1.7% procedural mortality	12% in historical controls
PH development	Potential protective effect	Emerging data needed	No clear association

Abbreviations: BPD, bronchopulmonary dysplasia; OR, odds ratio.

## 5 Evolution of Procedural Approaches

The report documents a significant shift in procedural approaches to PDA closure over recent years. Surgical ligation rates have declined substantially, from 8.4% to 2.9% between 2006 and 2015 among very low birth weight infants [1]. Simultaneously, transcatheter closure has gained popularity, with the Amplatzer Piccolo Occluder receiving FDA approval in 2019 for PDA closure in infants weighing ≥ 700 g. The authors provide a balanced assessment of both approaches. While surgical ligation achieves predictable closure, it carries risks, including vocal cord paralysis, pneumothorax, and potential associations, with increased BPD and neurodevelopmental impairment. Transcatheter closure has shown promising results, with high success rates (> 95% in recent studies) and relatively low complication rates, although the authors noted that experience and expertise vary among centers [1].

## 6 Gaps in Evidence and Future Directions

Despite extensive research, the report identifies significant knowledge gaps that require further investigation. The authors acknowledge that “there is a lack of evidence to guide management, necessitating equipoise regarding treatment options”. They highlight the need for well‐designed trials that evaluate not only ductal closure rates but also clinically critical long‐term outcomes. The ongoing PDA trial in the NICHD Neonatal Research Network and the PIVOTAL trial (Percutaneous Intervention *vs*. Observational Trial of Arterial Ductus in Low Birth Weight Infants) are mentioned as studies that may provide critical additional data to guide future recommendations [1].

## 7 Clinical Implications

For neonatologists and pediatric cardiologists, this report offers several practical takeaways:

1. A more selective approach to PDA management is warranted, with careful assessment of hemodynamic significance before intervention.
2. Conservative management is appropriate for many preterm infants, particularly in the first 2 weeks of life.
3. When intervention is deemed necessary, ibuprofen is generally preferred for medical closure, whereas transcatheter approaches are increasingly favored over surgical ligation for procedural closure.
4. Multidisciplinary collaboration between neonatologists and cardiologists is essential, particularly when considering procedural closure options.

## 8 Conclusion

This AAP clinical report thoughtfully synthesizes current evidence on PDA management in preterm infants. It acknowledges the complexity of the issue and the limitations of available data while providing practical guidance for clinicians. The shift toward more conservative management for many infants, coupled with selective intervention for those with hemodynamically significant PDAs that persist beyond the early neonatal period, reflects a more nuanced understanding of this common condition.

## Author Contributions

**Aditya Hemendra Bhatt:** conceptualization, formal analysis, writing – original draft, visualization. **Somashekhar Marutirao Nimbalkar:** conceptualization, formal analysis, writing – review and editing, supervision, validation. **Dipen Vasudev Patel:** visualization, writing – review and editing. **Reshma Kushal Pujara:** validation, writing – review and editing.

## Ethics Statement

The authors have nothing to report.

## Funding

The authors have nothing to report.

## Conflicts of Interest

The authors declare no conflicts of interest.

## Data Availability

No new data has been generated.

## References

[1] N. Ambalavanan , S. W. Aucott , A. Salavitabar , et al., “Patent Ductus Arteriosus in Preterm Infants,” Pediatrics 155, no. 5 (2025): e2025071425, 10.1542/peds.2025-071425. 40288780

[2] W. E. Benitz , C. O. F. A. Newborn , K. L. Watterberg , et al., “Patent Ductus Arteriosus in Preterm Infants,” Pediatrics 137, no. 1 (2016): e20153730, 10.1542/peds.2015-3730. 26672023

[3] R. I. Clyman , M. Liebowitz , J. Kaempf , et al., “PDA‐TOLERATE Trial: An Exploratory Randomized Controlled Trial of Treatment of Moderate‐To‐Large Patent Ductus Arteriosus at 1 Week of Age,” Journal of Pediatrics 205 (2019): 41–48.e6, 10.1016/j.jpeds.2018.09.012. 30340932 PMC6502709

[4] T. Hundscheid , W. Onland , B. van Overmeire , et al., “Early Treatment Versus Expectative Management of Patent Ductus Arteriosus in Preterm Infants: A Multicentre, Randomised, Non‐Inferiority Trial in Europe (BeNeDuctus Trial),” BMC Pediatrics 18, no. 1 (2018): 262, 10.1186/s12887-018-1215-7. 30077184 PMC6090763

[5] J. L. Bell , S. Gupta , E. Juszczak , P. Hardy , and L. Linsell , “Baby‐OSCAR: Outcome After Selective Early Treatment for Closure of Patent Ductus ARteriosus in Preterm Babies‐A Statistical Analysis Plan for Short‐Term Outcomes,” Trials 22, no. 1 (2021): 368, 10.1186/s13063-021-05324-3. 34039414 PMC8157743

